# Autoantibodies in the disease criteria for systemic sclerosis: The need for specification for optimal application

**DOI:** 10.1016/j.jtauto.2022.100141

**Published:** 2022-01-04

**Authors:** Jan Damoiseaux, Judith Potjewijd, Ruben L. Smeets, Carolien Bonroy

**Affiliations:** a Central Diagnostic Laboratory, Maastricht University Medical Center, Maastricht, the Netherlands; b Department of Internal Medicine, Division Nephrology and Clinical Immunology, Maastricht University Medical Center, Maastricht, the Netherlands; c Department of Laboratory Medicine, Radboudumc Laboratory for Diagnostics, Radboud University Medical Center, Nijmegen, the Netherlands; d Department of Laboratory Medicine—Medical Immunology, Radboud University Medical Center, Nijmegen, the Netherlands; e Department of Laboratory Medicine, Ghent University Hospital, Ghent, Belgium; f Department of Diagnostic Sciences, Ghent University, Ghent, Belgium

**Keywords:** Systemic sclerosis, Classification, Autoantibodies, Harmonization

## Abstract

The ACR/EULAR classification criteria for systemic sclerosis (SSc) entail three autoantibodies: anti-centromere antibodies (ACA), anti-topoisomerase I antibodies (ATA), and anti-RNA-polymerase III antibodies (ARA). The importance of ACA and ATA in the classification criteria is evidence based, but the diagnostic value is overestimated by clinicians. Fortunately, these autoantibodies are characterized by good agreement between different immuno-assays. Inclusion of ARA, however, is based on limited evidence and is related to limited agreement between different immuno-assays. Harmonization of immuno-assays in terms of interpretation based on likelihood ratio's may improve future classification criteria for SSc and this needs to be achieved by close collaboration between clinicians, laboratory specialists and the diagnostic industry.

## 1 Introduction

Systemic sclerosis (SSc) is a severe disease within the spectrum of the systemic autoimmune rheumatic diseases (SARD) that are differentially associated with autoantibodies. Multiple autoantibodies can be found in SSc and they are helpful for diagnosis, for classification of disease subtypes and even may be prognostic for disease progression, organ involvement and/or development of malignancies [1]. However, not all of these autoantibodies have the proper test-characteristics that make them robust enough to be included in classification criteria.

The first preliminary criteria for the classification of SSc (1980) were defined by the subcommittee for scleroderma criteria of the American Rheumatism Association Diagnostic and Therapeutic Criteria Committee [2]. These criteria already recognized the value of autoimmune serology based on the use of HEp-2 indirect immunofluorescence assays (IIFA) to detect the anti-centromere (ACA) and anti-topoisomerase I (ATA; formerly anti-Scl70) antibodies. At that time, the basic, heat-labile, chromatin-associated, nonhistone 70 kD protein was only very recently (1979) identified as the target of autoantibodies in SSc [3].

About 20 years later (2001), the LeRoy & Medsger criteria were defined for classification of early systemic sclerosis [4]. These included a wider set of SSc-associated autoantibodies, including ACA, ATA, anti-fibrillarin (anti-U3RNP), anti-polymyositis-scleroderma (PM-Scl), anti-fibrillin, and anti-RNA polymerase III (ARA) antibodies. The scientific background for including these autoantibodies and information about the preferred method of detection was not provided, except that antibodies should have a titer of at least 1:100 and the reference to the work of Eng Tan [5]. This extensive review, however, only described the association of these autoantibodies with SSc, but did not provide evidence for the 1:100 titer in relation to the respective autoantibodies. Subsequently, the criteria for early systemic sclerosis were validated [6]. Although this study more extensively described the methods used for autoantibody detection, is has not been established if other immuno-assays perform equally good.

Finally, in 2013 a collaborate action between the American College of Rheumatology (ACR) and European League Against Rheumatism (EULAR) resulted in a new set of classification criteria for SSc [7]. These criteria were established by a pre-defined protocol for designing disease criteria. These criteria include the restricted number of three different SSc-related autoantibodies, i.e., ACA, ATA, and ARA. The presence of these antibodies can be determined by a wide variety of immuno-assays that may differ in terms of test-characteristics due to differences in, among others, antigen exposure, detection method, cut-off setting, testing algorithm, and reporting of results. While the criteria focused on the definitions of the clinical manifestations, the possible influence of the differences in immuno-assays were largely overlooked.

Although classification criteria are not designed for diagnostic purposes, it should be kept in mind that in the absence of diagnostic criteria, the classification criteria will be in the mind of clinicians during the diagnostic work-up of patients with SSc in the differential diagnosis. Therefore, it is important to harmonize the outcome and interpretation of the immuno-assays as far as possible [8], in order to incorporate diagnostic test specifications for autoantibody detection in future disease classification criteria.

## 2 Test-characteristics of autoantibodies included in the SSc classification criteria

In general, test-characteristics are defined by the cut-off value of the immuno-assay, in order to distinguish between negative and positive, in combination with the disease and control population selected for discrimination between true and false results. The setting of the cut-off is most often defined by the diagnostic company and is based on different choices [9]. As such, this item is prone to harmonization. Furthermore, both the disease and control population should represent the patient population that is being tested for the respective marker. For SSc this implies that the different SSc subsets should be represented according to prevalence in the population and that controls should include patients that present with clinical manifestations mimicking SSc. Any bias in the inclusion will affect the test-characteristics and hamper appropriate interpretation of the test results.

In 2003 evidence-based guidelines for the use of ACA and ATA were published by the ACR [10]. An extensive literature review revealed the test-characteristics for these autoantibodies in relation to method of detection. The sensitivity of ACA as determined by IIFA was 31.9% (range 2–59%), while the specificity was 99.9% (range 99.8–100%) as compared to healthy controls and 97.0% (range 83.4–100%) as compared to other SARD. The sensitivity of ATA as determined by immunodiffusion was 25.1% (range 17–67%), and the specificity 100% (for all studies) and 99.5% (range 97.8–100%) as compared to healthy controls and other SARD, respectively. If the ATA were determined by enzyme-linked immune-sorbent assays (ELISA; only two studies) the sensitivity was 43.5%, and the specificity 100% and 89.6%, respectively. The test-characteristics of ARA were only poorly defined because only a limited number of studies was available at that time, i.e., 2003. Moreover, a clear distinction between antibodies to RNA-Polymerase I, II, and III was limited and assays were primarily based on immunoprecipitation and immunoblotting, i.e., assays that are not readily available in routine clinical laboratories.

A systematic review and meta-analysis on the prevalence of ARA was published by Sobanski et al. in 2013 [11]. The overall pooled prevalence of ARA was 11% (95% CI: 8–14), but with a range of 0–41%. The heterogeneity among studies was primarily explained by geographical factors, but it was also apparent that a wide diversity of immuno-assays was used in the 30 studies included. In the review of Mehra et al. the prevalence of ARA in SSc was reported to be about 20%, again with differences between geographical continents [12]. Both studies did not report the specificity of this biomarker.

A more recent review summarized the test-characteristics of autoantibodies in SARD, including SSc [13]. In this review the prevalence of ACA, ATA and ARA were reported to vary between 28.2 and 36.9%, 30.1–41.2% and 3.8–19.4%, respectively. Separately, data for sensitivity (44%, 43% and 38%, respectively) and specificity (93%, 90% and 94%, respectively) were provided. The latter data were reported to be derived from the ACR study by Reveille et al. [10]. Interestingly, the data for sensitivity are not within the reported range for prevalence, while these parameters are basically the same. Furthermore, the data for sensitivity and specificity are incompletely and even incorrectly interpreted. In particular the sensitivity (38%) and specificity (94%) of ARA as provided by Reveille et al. are not diagnostic characteristics, but are about the prognostic value for cutaneous involvement in SSc patients.

Altogether, it is evident that there is wide variety between studies with respect to reported test-characteristics for the three SSc-associated autoantibodies that are included in the ACR/EULAR classification criteria. While this can be attributed to geographical and ethnical differences, the type of assay used as well as the comparator population (healthy controls *versus* disease controls) will also impact on the outcome. Moreover, invalid interpretation and combination of study data will further add to the poorly defined test-characteristics for these autoantibodies. This may have affected the anticipated value of autoantibody results by clinicians involved in the generation of classification criteria.

## 3 Scientific background for inclusion of autoantibodies in the ACR/EULAR classification criteria

To identify items relevant for the revised classification criteria for SSc, first a consensus exercise was performed based on standard consensus procedures [14]. Starting with 168 potential items, three subsequent rounds of Delphi-scoring by experts in the field eventually revealed 23 items, each with an appropriateness score (1–9) and ranking in relation to the other 22 items. Within the set of 23 items, five items involved autoantibodies: ACA, ATA and ARA all had the highest appropriateness score (9) and the ranks 3, 2 and 6, respectively. Autoantibodies to PM-Scl and anti-nuclear antibodies (ANA) had a rather low appropriate score (5) and a ranking of 20 and 13.

Next, validation of the respective autoantibodies was performed in five well-defined patient cohorts originating from North-America and Europe [15]. Sensitivity and specificity of the five autoantibodies is shown in Table 1. Importantly, the comparator population greatly differed between the cohorts. For instance, the Canadian Scleroderma Research Group cohort was compared with a Lupus cohort, while the Berlin cohort did not include any comparator population. The first choice, obviously, has a huge impact on the specificity of ANA (2%), while the latter choice hinders appropriate interpretation of the test-characteristics. Furthermore, complete information to determine both sensitivity and specificity for ARA and anti-PM-Scl was only available from the Pittsburgh Connective Tissue Disease cohort. Considering the wide heterogeneity in test-characteristics between studies, it is questionable if these data are representative for other cohorts. The positive likelihood ratio (LR+) for ARA in the Pittsburgh Connective Tissue Disease cohort is 26 (reported OR is 75.4 with a 95% CI of 13.2–312.6), while LR+ for anti-PM-Scl is only 1.5 (OR: 2.4; 95% CI: 1.9–7.1). Information about the immuno-assays used for the detection of the autoantibodies is not provided, but may be available in the original studies describing these cohorts. Based on pooled ORs the 23 candidate criteria were ranked and data were compared with the expert-based ranking. Empirical ranking was the highest for ARA (4) and the lowest for anti-PM-Scl (19). Interestingly, expert-based ranking for ATA (2) and ACA (3) was much higher than empirical ranking (8 and 11, respectively).

**Table 1:** Test characteristics of autoantibodies in 5 well defined cohorts used for empirical ranking of criteria.

Cohort^a^	# Patients		ACA		ATA		ARA		PM-Scl		ANA	
	# SSc	# Controls	sens	spec	sens	spec	sens	spec	sens	Spec	sens	spec
**CSRG**^b^**cohort**	**127**	**127** (SLE)	**29**	**99**	**17**	**99**	**18**	*NA*	**11**	*NA*	**93**	**2**
**Pittsburg CTD cohort**	**326**	**327** (SLE/IIM/SjS)	**30**	**95**	**20**	**98**	**26**	**99**	**3**	**98**	**95**	**23**
**Toronto cohort**	**86**	**114** (SLE, MCTD, PAH)	**16**	**96**	**17**	**99**	*NA*	*NA*	*NA*	*NA*	*NA*	*NA*
**Madrid cohort**	**175**	**411** (SLE/IIM/RP)	**27**	**100**	**35**	**99**	*NA*	*NA*	*NA*	*NA*	**94**	**38**
**Berlin cohort**	**69**	**0** (not applicable)	**28**	*NA*	**22**	*NA*	**6**	*NA*	**4**	*NA*	**91**	*NA*

a For details see Johnson et al. [15].

b Abbreviations: ACA, anti-centromere antibodies; ANA, anti-nuclear antibodies; ARA, anti-RNA polymerase III antibodies; ATA, anti-topoisomerase I antibodies; CSRG, Canadian Scleroderma Research Group cohort; IIM, idiopathic inflammatory myopathy; MCTD, mixed connective tissue disease; NA, not available; PAH, pulmonary arterial hypertension; PM-Scl, anti-polymyositis-scleroderma antibodies; RP, Raynaud phenomenon; sens, sensitivity; spec, specificity; SjS, Sjögren's syndrome; SLE, systemic lupus erythematosus; SSc, systemic sclerosis.

Finally, a multi-criteria additive point system was evaluated in a derivation cohort and confirmed in a validation cohort [7]. The test-characteristics for the three included autoantibodies are provided in Table 2. Especially when expressed as LR+ an enormous difference becomes apparent between the derivation and validation cohort. The underlying reason for this difference is not further explored or discussed, possibly because the overall disease criteria show less difference in terms of sensitivity and specificity between the derivation and validation cohort. In the resulting classification system a total score of ≥9 represents a definite SSc classification. The presence of ACA, ATA and/or ARA represents a score of 3 (maximum score is 3). The only additional information about the autoantibodies includes that ACA can be defined in solid-phase immuno-assays or by a centromere pattern in the HEp-2 IIFA. Furthermore, a positive result is to be defined according to local laboratory standards.

**Table 2:** Test characteristics of autoantibodies in derivation and validation cohort of ACR/EULAR classification criteria.

Cohort^a^	# Patients		ACA^b^			ATA			ARA		
	# SSc	# Controls	sens	spec	LR+	sens	spec	LR+	sens	spec	LR+
**Derivation cohort**	**100**	**100**	**33**	**95**	**6.7**	**34**	**99**	**34**	**2**	**99**	**2.0**
**Validation cohort**	**268**	**137**	**15**	**94**	**2.5**	**26**	**95**	**5.2**	**10**	**100**	**∞**

a For details see Van den Hoogen et al. [7].

b Abbreviations: ACA, anti-centromere antibodies; ARA, anti-RNA polymerase III antibodies; ATA, anti-topoisomerase I antibodies; LR+, positive likelihood ratio; sens, sensitivity; spec, specificity; SSc, systemic sclerosis.

## 4 Influence of different methods and/or assays

Evidently, for the autoantibodies the classification criteria do not take into account the differences in methods and reporting of results. There are many studies that have compared different immuno-assays for the detection of the respective autoantibodies, but here we will focus on two studies only. The first compared two line-immuno-assay (LIA; Systemic Sclerosis Profile [Euroimmun] and INNO-LIA ANA Update [Innogenetics]; the latter only contains CENP-B and DNA topoisomerase I) with a combination of more conventional techniques that are often considered gold standard, including HEp-2 IIFA, Western blotting, protein immuno-precipitation, and double immuno-diffusion [16]. A more recent study compared three assays widely used in clinical practice, *i.e.*, Fluorescent-Enzyme Immuno-Assay (FEIA; ThermoFischer), LIA (Euroimmun), and dot-immuno-assay (DIA; Dtek) [17].

For ACA the option is included to detect these antibodies either by IIFA or solid-phase immuno-assays. According to the international consensus on ANA patterns (ICAP) [18], a low titer centromere pattern (AC-3) is to be confirmed by an antigen-specific immuno-assay for anti-CENP-B [19]. This indicates that, together with the pattern, the titer should be part of the report of the HEp-2 IIFA result [20]. However, the centromere pattern is most often associated with a high titer [21,22]. There is no information whether anti-CENP-A is also sufficient to fulfill the criteria, but in case of ACA there is a very high consistency between assays for the HEp-2 IIFA centromere pattern (AC-3), CENP-A reactivity and CENP-B reactivity. As compared to conventional techniques, i.e., IIFA and western blotting with nuclear extracts (72 positive cases in a cohort of 145 SSc patients), the combined presence of anti-CENP-A and -B on the Euroimmun LIA (72 positive cases in a cohort of 145 SSc patients) revealed very good agreement between methods (κ = 0.820) [16]. The Innogenetics LIA showed very good agreement for CENP-B with the Euroimmun LIA (κ = 0.888) [16]. In the study of Alkema et al. all three assays enabled detection of anti-CENP-B, while the DIA and LIA also enabled detection of anti-CENP-A. Positive results ranged from 99 to 105 patients in a cohort of 347 SSc patients; there was an almost perfect agreement between the distinct assays, even between anti-CENP-A and CENP-B (κ-range 0.96–0.99) [17].

ICAP also has defined a related pattern for ATA (Topo I-like; AC-29), but this pattern is more difficult to recognize (expert level) and always requires confirmation by an antigen-specific immuno-assay [23]. Less experienced laboratories most likely report a nuclear homogeneous pattern (AC-1). The concordance between different antigen-specific immuno-assays is also very high for ATA, but somewhat less than for ACA. In the study of Bonroy et al. results of the Euroimmun LIA (28 positive cases in a cohort of 145 SSc patients) were compared with double immunodiffusion (26 positive cases in a cohort of 145 SSc patients) as conventional technique and revealed very good agreement (κ = 0.909) [16]. The Innogenetics LIA also showed very good agreement for ATA with the Euroimmun LIA (κ = 0.885) [16]. This was confirmed in the second study where a range of 72–83 patients tested positive in the cohort of 347 SSc patients (κ-range 0.91–0.94) [17].

In case of ARA the situation is more complex. First, the associated HEp-2 IIFA pattern, i.e., large/coarse speckled (AC-5), is far from specific for RNA-Polymerase III [19]. Second, there is substantial variation in the antigen composition of immuno-assays. Some assays contain both RP11 and RP155 as separate entities, while others contain only the RP155 antigen. In particular if two antigens are available, interpretation may be a challenge. As compared to the conventional detection technique, i.e., protein immunoprecipitation, results of the Euroimmun LIA (11 positive cases in a cohort of 145 SSc patients) were in perfect agreement (κ = 1.000) if both RP11 and RP155 were positive; almost perfect agreement (κ = 0.831) was obtained if at least one of the antigens was recognized in the LIA [16]. The study of Alkema et al. revealed more heterogeneity between assays (10–24 positive cases in a cohort of 347 SSc patients) varying from moderate to substantial agreement (κ-range 0.53–0.76) depending on which assays were compared to each other [17]. Even if assays were only compared for reactivity to RP155, the range in κ-values remained similarly large [17].

## 5 Influence of testing algorithms in routine clinical practice

Different testing strategies are being applied in routine clinical laboratories for the detection of autoantibodies included in the classification criteria for SSc. It is well appreciated that HEp-2 IIFA is a valid screening method for these antibodies [24]. As mentioned, this method also enables to directly identify ACA without the further need for confirmation. Alternatively, solid-phase immuno-assays are increasingly used to screen for SARD-associated autoantibodies. The antigenic composition of these assays is highly variable [25]: some lack the CENP-B antigen and many lack the RNA-polymerase III antigen(s). The lack of the CENP-B antigen is less of a problem as long as the solid-phase immuno-assay is used in parallel with the HEp-2 IIFA, but there is no good back-up for detection of the ARA.

After all, the decision to not include the HEp-2 IIFA in the classification criteria, perhaps should be revisited. As already mentioned, the ANA did not have a high empirical ranking because the comparator populations also had high prevalence of ANA, resulting in a very low specificity for SSc [15]. However, SSc has a very high prevalence of ANA, as detected by HEp-2 IIFA, and therefore a positive HEp-2 IIFA result could be used as an entry criterion, similar to the recent ACR/EULAR criteria for SLE [26]. For SSc an alternative to the HEp-2 IIFA, probably, is not appropriate because in particular the nucleolar antigens are only poorly represented in the solid-phase screening assays. Anyway, a positive ANA screen result is to be followed by reflex testing, either automatic or upon request, in order to determine antigen-specificity of the autoantibodies. Clinical laboratories use different algorithms that may impact on finding the relevant SSc-associated autoantibodies. After ANA positivity, some laboratories first analyze the sample for the presence of autoantibodies to RNP, Sm, Ro/SSA (Ro60 and/or Ro52), and La/SSB; if positive, no further testing is performed. Since anti-Ro52 antibodies are quite common in SSc, especially in combination with CENP-B, this strategy may prevent from finding the clinically more relevant autoantibody. Again, many assays used for determining the antigen-specificity lack the RNA-polymerase III antigen(s). Obviously, upon clinical suspicion of SSc a so-called scleroderma autoantibody profile can be requested to close this gap [27], but this assay may only be available in specialized laboratories. Furthermore, clinicians should take into account that testing algorithms are often defined for the whole spectrum of SARD. If testing for ARA is broadly included in these algorithms, this will increase the ratio of false- and true-positive results [28].

## 6 Conclusion

The three autoantibodies that are part of the ACR/EULAR classification criteria for SSc, i.e., ACA, ATA, and ARA, were included based on a combination of expert opinion and data obtained from five SSc study cohorts. Empirical ranking revealed that for the diagnostic value of these autoantibodies expert opinion was over-estimated [14,15]. Furthermore, empirical ranking of ARA was based on data from a single cohort. The final classification criteria were, subsequently, evaluated in a derivation and validation cohort. Although the test-characteristics for all three autoantibodies strongly differed between both cohorts, this was not further elaborated upon in the publication on the 2013 classification criteria [7].

Evidently, the classification criteria could be further improved by harmonization of the respective immuno-assays in terms of optimal cut-off settings and interpretation of results. Although for ACA a very high agreement between assays was observed, this can be further improved by defining if the HEp-2 IIFA centromere pattern is to be confirmed in antigen-specific immuno-assays, and if yes, whether that only includes reactivity to CENP-B, or also CENP-A. The agreement for ATA is also very good; interpretation of the test-results for these antibodies may benefit from confirmation in an independent antigen-specific immuno-assay, in particular in patients with doubtful clinical manifestations. The third autoantibody, ARA, however, requires further attention. Exclusion from the criteria might be considered unless more empirical data support the inclusion. The doubtful inclusion of ARA is best illustrated by the extreme differences in LR+ between the derivation and validation cohorts and the impact of the selected assay on the test-result [7,17]. In addition, also other SSc-associated autoantibodies should be further explored for added value in the classification criteria. However, prevalence of these autoantibodies is generally rather low and, similar to the situation in idiopathic inflammatory myopathies [29], this hampers the generation of solid data-sets to support inclusion. Furthermore, also assays for detection of autoantibodies to antigens like fibrillarin and Th/To are highly variable in terms of outcome.

In contrast to many laboratory tests, there is no good option for standardization of immuno-assays for autoantibodies [7]. Harmonization, seems to be the best alternative. One reason for the observed heterogeneity in test-results is due to different approaches used by the diagnostic industry to define the optimal cut-off for the assay [9]. This can be aligned by defining a cut-off, based on a certain level of specificity, for instance 95% or 98%. Alternatively, test-results could be expressed as likelihood ratio, either for test-result-intervals, or even for single test-results [30]. This approach gives credit to the level of autoantibody that is being evaluated and could contribute to a more elegant scoring system as compared to the “3 points for all” in the current classification system.

In the end, this contribution hopefully paves the road for active collaboration with clinicians, laboratory specialists and the diagnostic industry with experience in autoantibody testing in better defining the laboratory parameters in future classification criteria for SSc.

## Declaration of competing interest

The authors declare that they have no known competing financial interests or personal relationships that could have appeared to influence the work reported in this paper.
